# A prospective multi-center study quantifying visual inattention in delirium using generative models of the visual processing stream

**DOI:** 10.1038/s41598-024-66368-4

**Published:** 2024-07-08

**Authors:** Ahmed Al-Hindawi, Marcela Vizcaychipi, Yiannis Demiris

**Affiliations:** 1 Personal Robotics Laboratory, Department of Electrical and Electronic Engineering, Imperial College London, London, SW7 2AZ UK; 2 Department of Anaesthesia, Pain Medicine and Intensive Care, Chelsea and Westminster Hospital NHS Foundation Trust, London, SW10 9NH UK

**Keywords:** Neurodegenerative diseases, Data processing, Image processing

## Abstract

The visual attentional deficits in delirium are poorly characterized. Studies have highlighted neuro-anatomical abnormalities in the visual processing stream but fail at quantifying these abnormalities at a functional level. To identify these deficits, we undertook a multi-center eye-tracking study where we recorded 210 sessions from 42 patients using a novel eye-tracking system that was made specifically for free-viewing in the (ICU); each session lasted 10 min and was labeled with the delirium status of the patient using the Confusion Assessment Method in ICU (CAM-ICU). To analyze this data, we formulate the task of visual attention as a hierarchical generative process that yields a probabilistic distribution of the location of the next fixation. This distribution can then be compared to the measured patient fixation producing a correctness score which is tallied compared across delirium status. This analysis demonstrated that the visual processing system of patients suffering from delirium is functionally restricted to a statistically significant degree. This is the first study to explore the potential mechanisms underpinning visual inattention in delirium and suggests a new target of future research into a disease process that affects one in four hospitalized patients with severe short and long-term consequences.

## Introduction

The visual processing deficits underlying visual inattention in delirium remain poorly understood despite visual inattention forming a crucial part of the definition of delirium^1^. Identifying these deficits is crucial for the understanding of the pathogenesis of delirium and for the development of targeted therapies in a syndrome that affects one in four hospitalized individuals with its associated mortality, morbidity, and long-term cognitive sequelae^2–7^. Radiological investigations, including Magnetic Resonance Image (MRI) and Computed Tamography (CT) Positron Emission Tomography (CT-PET), have revealed evidence of functional disconnectivity between the occipital lobe, specifically the ventral visual processing areas, and the anterior cingulate cortex, a region crucial for memory and attention regulation^8–10^. Similarly, Electroencephalogram (EEG) studies have demonstrated the diagnostic specificity of electrodes located on the occipital lobe, implicating altered neuronal responses in visual processing as a potential core feature of delirium^5,6^. However, despite these valuable insights, current Radiological and EEG approaches fall short of providing a comprehensive quantification of the functional errors in visual processing that occur in patients with delirium.

The definition of attention in the delirium literature does not draw distinction on the type of attention. and we therefore chose to focus on visual attention as a physically measurable signal that manifests from internal cognitive functions^11,12^. Broadly, cognitive attention refers to the capacity to maintain focus on a particular mental task or thought, involving the brain’s executive control systems that manage sensory inputs and coordinate responses^13^. This form of attention is integral to memory, decision-making, and problem-solving. A subset of this is visual attention; this type of attention involves mechanisms for detecting and responding to visual elements within the environment, which are intrinsicly linked to higher cognitive processes^14^. In the context of delirium, quantifying and understanding visual attention is is pivotal to progress the understanding of the specific pathways and mechanisms affected by delirium.

To examine and quantify the visual processing that patients with delirium exhibit that in could result in visual attention deficits, we deplyed a novel camera-based eye-tracking device prospectively across two Intensive Care Units (ICUs) general district hospitals. We recorded 262 eye-tracking sessions across 42 patients (ClinicalTrials.gov—NCT04589169) where the patients were free to view the scene in front of them without specific stimulus. Each recording was labeled with the patient’s delirium status using Confusion Assessment Method in ICU (CAM-ICU)^12^.

We then developed a novel neurocomputational generative model capable of simulating and comparing functional units of the ventral visual processing stream. This model leverages the functional hierarchy of visual processing to emulate neural processes given the scene that is being viewed and an internal cognitive task. This enables the creation of a dynamic baseline for visual attention. These models simulate the typical visual processing pathway, dynamically adapting to changes in the visual scene and the viewer’s fixation and subsequently, their attention. By modeling the normal hierarchical processes of visual attention-from basic visual cue detection in early units to complex object and face recognition in later stages-we establish what normal attentional engagement should look like across different visual tasks and environments. This baseline model can then facilitate comparisons between different patients by constraining the comparison to that baseline—our generative model.

We validate our generative model in two ways, firstly under simulation to understand its characteristics, and secondary in an existing dataset of participants undergoing a visual search task. We then apply the model to the eye-tracking data collected from patients in Adult Intensive Care Unit (AICU) to quantify the functional errors in visual processing that occur in patients with delirium.

The data and the analysis technique suggest that delirium is punctuated by functionally restricted processing throughout the visual processing hierarchy. This finding quantifies this restriction and posits that a potential mechanism that underlies visual in-attention could be at the level of visual encoding prior to working memory, as previously thought.

## Method

The reported study was approved by the NHS Health Research Authority (HRA) and Research & Ethics Committee (REC) (20/LO/0162) and registered on ClinicalTrials.gov (NCT04589169^15^). As delirium is a capacity-losing state, informed consent was sought from a next of kin or a nominated consultee should the patient suffer from delirium. Patients were recruited between November 2020 and February 2022 from two interdisciplinary general medical and surgical hospitals. Participants were not remunerated for their participation in the study. All research methodology was conducted in accordance with the Declaration of Helsinki.

**Figure 1:**
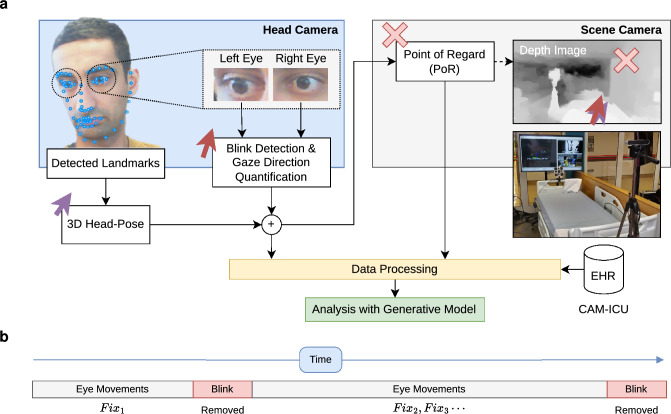
Illustration of the camera setup and how gaze vectors were extracted to be converted to fixations. The head camera is placed facing the patient and is used to regress the gaze vector through an ensemble of neural networks. The scene camera is placed behind the patient and is used to intersect the gaze vector with the scene revealing the pixel location of the patient’s gaze, termed the Point of Regard (PoR). The data generated is then stored, labeled with the patient’s delirium status, and analyzed.

### Participants

A total of 42 patients were recruited who had a total risk-adjusted probability of developing delirium greater or equal to 20% as per the Early PREdiction of DELIRium in ICu patients (E-PRE-DELIRIC) score^16^. Exclusion criteria were limited to the inability to measure eye movements appropriately or gain ground truth annotation of delirium. Delirium was diagnosed by fluctuating alteration in cognition and memory formalized through CAM-ICU which was performed by an Intensive Care clinician contemporaneously with the measurements using the apparatus^12^. Participants were recruited from ICUs from Chelsea & Westminster Hospital (CWH) and West Middelsex Hospital (WMH) and NHS approvals, two district hospitals recieving general medical and surgical patients. Briefly, were sequentially recruited into the study as they were admitted to ICU and they met the eligbility criteria; Supplementary Information A details the recruitment process. Table 1 lists the characteristics of the patients enrolled in the study.

**Figure 2:**
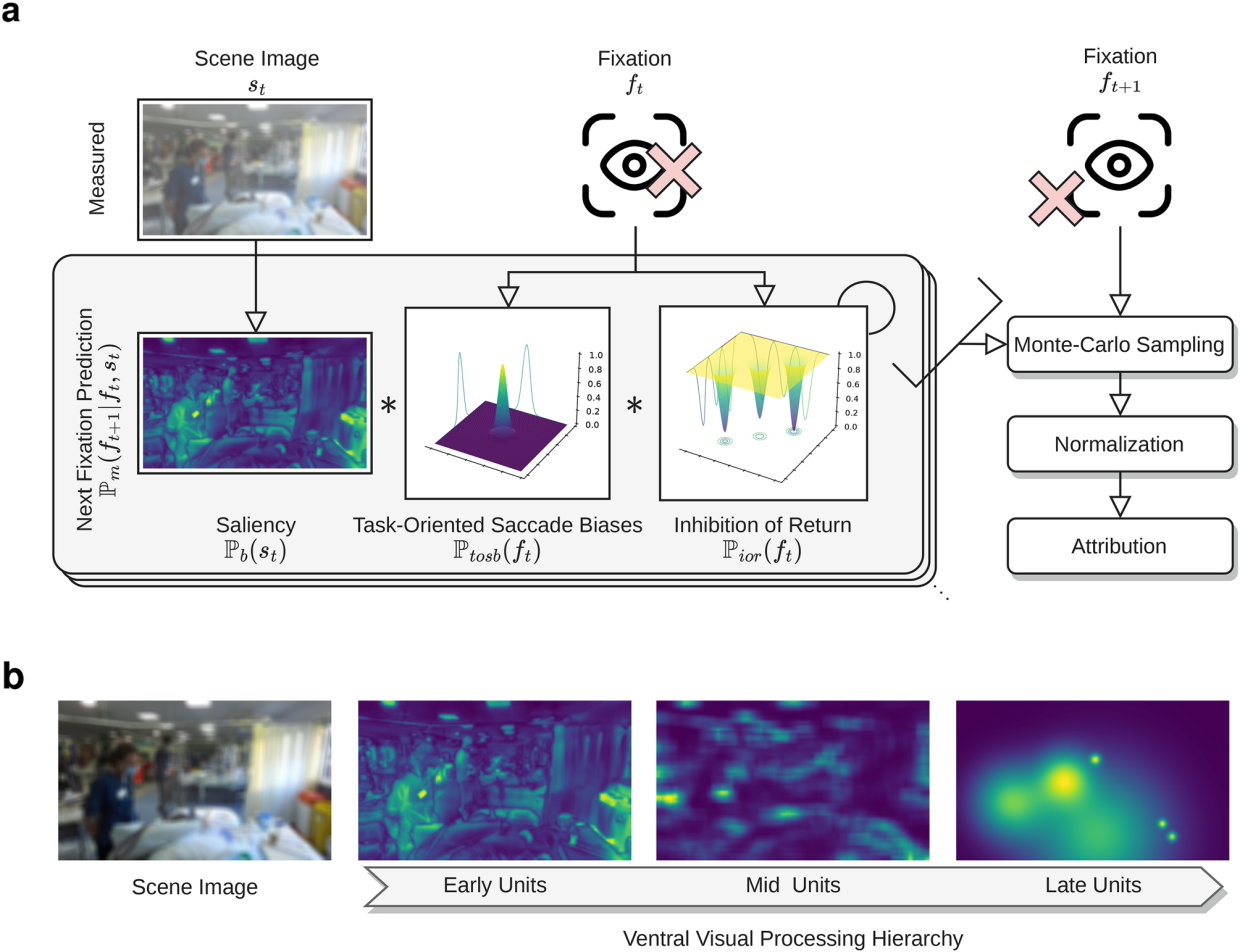
Illustration of the generative model, composed of a hierarchical set of models, that output a probabilistic map of fixation locations that can then be compared to patient fixations to infer the function of the visual processing stream. Each model is composed of saliency, task-oriented saccade biases, and inhibition of return and they jointly result in a probabilistic map of the future fixation. The error of this prediction, compared to the actual measurement ($f_{t+1}$) can then be calculated, and normalized, and the model with the highest probability wins a token. These are then tallied up and compared. Saliency is calculated from the scene information which forms bottom-up signal propagation whilst the inhibition of return and saccade biases model top-down influences on eye-movement behavior.

### Eye movements & fixation measurement apparatus

A dual camera system was developed and validated for the exact purpose of eye movement measurement in patients in ICU in a clinically safe, accurate, and calibration-free manner^15,17^. Briefly, one camera is placed facing the patient, at a distance that does not interfere with clinical duties, and continuously regresses the gaze vector through an ensemble of neural networks. These gaze vectors were converted to fixations using a dispersion filter—the sequence of gaze vectors is classified as a fixation if their dispersion falls below $6^{\circ }$ and lasted between 300 and 600 ms^18,19^. Another, depth enabled, camera is then located behind the patient and looks at the scene with the same field of view as the participant. This camera is used to intersect the fixation vector with the scene revealing the pixel location of the patient’s gaze^20^. The apparatus is placed for 10 min daily until discharge, with concurrent CAM-ICU measurement. The participants were not asked to perform any specific task while the gaze measurements were taken using the apparatus. Fig. 1 illustrates the apparatus.

### Generative model overview

As the task of comparing eye movements from recordings of patients undergoing natural stimuli is unconstrained, we sought to create a generative baseline that can serve as a comparator. We empirically chose to create a neuro-computational baseline where we define the task of fixation prediction as a composition of a top-down, task-dependent signal, a temporal short-term memory component, and a bottom-up, scene-dependent signal^21–25^. This is formalized into a model that can generate a distribution over the set of future fixations:

1 $$\begin{aligned} \mathbb {P}_{m}(f_{t+1}|f_t,s_t) = \underbrace{\mathbb {P}_{b}(s_t)}_\text {Bottom Up} \odot \underbrace{\mathbb {P}_{tosb}(f_t) \odot \mathbb {P}_{ior}(f_t)}_\text {Top Down} \forall m \in \mathscr {M} \end{aligned}$$

where $\odot$ is the element-wise product, $\mathbb {P}(f_{t+1}|f_t,s_t)$ is the probability distribution of the next fixation $f_{t+1}$ conditional on the current fixation *f* and the scene *s* at time *t*. Task-oriented saccade biases, $\mathbb {P}_{tosb}$, encode a joint probability of saccadic amplitude and direction which are known to be task dependent^26^. $\mathbb {P}_{ior}$ represents the probability of returning to a fixated location within a short period and functionally represents short-term memory, facilitating the exploration of the scene without return within *T* fixations:

2 $$\begin{aligned} \mathbb {P}_{ior}(f_t)\!=\!\left\{ \!\begin{array}{cc} 1,&{}t = 0, \\ \!\left\lfloor \mathbb {P}_{ior(f_{t\!-\!1})}-\Phi _i+\sum \limits _{k=1}^{\xi }{\frac{\Phi _r}{T}}\right\rfloor &{}\!\!\text {otherwise}, \forall y \in \mathbb {I} \end{array}\right. \end{aligned}$$

where $\Phi$ represents a Gaussian distribution parameterized by a standard deviation set to $2^\circ$ reflecting the width of the human fovea, $\Phi _i$ is the inhibition Gaussian defined as $\Phi (\Vert y-f_t\Vert _2)$ and $\Phi _r$ is the recovery Gaussian defined as $\Phi (\Vert y-f_{t-k}\Vert _2$. $y \in \mathbb {I}$ represents all locations in image $\mathbb {I}$. $\Vert .\Vert _2$ is the Euclidean distance, $\left\lfloor . \right\rfloor$ clips the values between [0, 1]. $\xi$ is the number of fixations to consider and is equal to $min(T, t-1)$ where *T* is nominally set to 8^24^. This function is effectively discouraging return of the fixation to the same location within a short period, with a decaying profile.

Bottom-up fixations are modeled using a saliency map where $\mathbb {P}_{b}$ is the probability of a fixation given the intrinsic parameters of that saliency model of the scene. We chose saliency models that represent the hierarchy of the visual processing stream starting from simple geometric shapes and increasing in complexity up to object-level saliency.

The combination of $\mathbb {P}_{b}$, $\mathbb {P}_{ior}$, and $\mathbb {P}_{tosb}$ represent one model *m* from a set of models $\mathscr {M}$ each generating a distribution of the next fixation. We then compose multiple models, each with a different intrinsic notion of task-oriented saccade biases, inhibition of return, and saliency. The *correctness* of each model can then be calculated by sampling from the resulting distribution over the predicted fixation ($\mathbb {P}_m$) using the distance to the *actual* next fixation as the metric:

3 $$\begin{aligned} d_{m}(fix_{t+1}) = \mathbb {E}_{n \sim \mathbb {P}_{m}}\bigg [\frac{\Vert n - fix_{t+1}\Vert _{2}}{diag(\mathbb {I})}\bigg ] \forall m \in \mathscr {M} \end{aligned}$$

where $\mathbb {E}$ is the expectation operator that samples *n* from the probability distribution for model $\mathbb {P}_{m}$. $\Vert *\Vert _2$ is the $\ell _2$ norm. To normalize these measurements, the distances are normalized to the maximum possible value—the diagonal of the scene image $\mathbb {I}$. This results in a distance metric where smaller distances are a result of more accurate predictions for that model. As smaller distances between the simulated fixation and the measured fixation, the following softmax function is used:

4 $$\begin{aligned} \sigma _m = \frac{e^{-\beta (1-d_{m})}}{\sum _{j=1}^{|\mathscr {M}|} e^{-\beta (1-d_j)}} \forall m \in \mathscr {M} \end{aligned}$$

where $d_m$ is the current model’s distance, and $|\mathscr {M}|$ is the cardinality of the set of all models. Parameter $\beta$ dictates the sharpness of the softmax function encouraging separation between models. This results in a normalized distribution where the model that is most in line with measured fixation ($fix_{t+1}$) has the highest probability.

**Table 1:** Characteristics of patients enrolled as part of the deployment of the eye-tracking platform forming a clinical feasibility study. Pertinent confounding variables related to outcomes of interests as well as variables that predict the development of delirium are presented across the two hospitals; Chelsea & Westminster Hospital (CWH) and West Middelsex Hospital (WMH).

	Chelsea & Westminster Hospital (CWH)	West Middelsex Hospital (WMH)	Overall
Sessions	186	76	262
Patients	32 (77.1%)	10 (22.9%)	42
CAM-ICU			
Positive	75	39	114
Negative	69	27	96
Unable to measure	42	10	52
Gender			
Female	11	4	15
Male	21	6	27
Age (years)			
Mean ± standard deviation	57.3 ± 19.3	58.0 ± 14.7	57.5 ± 18.1
Frailty score			
Mean ± standard deviation	3.48 ± 1.05	1.50 ± 0.53	3.03 ± 1.27
APACHE-II probability (%)			
Mean ± standard deviation	23.5 ± 14.1	29.5 ± 20.7	24.9 ± 15.7
Urea (mmol/L)			
Mean ± standard deviation	10.9 ± 7.08	10.8 ± 5.96	10.9 ± 6.76
Corticosteroids			
No	31	10	41
Yes	1	0	1
Admission type			
Elective	8	2	10
Emergency	24	8	32
ICU length of stay (days)			
Mean ± standard deviation	22.2 ± 25.9	27.3 ± 24.1	23.4 ± 25.2
Unit outcome			
Alive	25	8	33
Dead	7	2	9

Each model *m* then competes for attribution in a winner-takes-all scheme where the model with the highest probability ($\sigma _m$), and thus the lowest error, is rewarded a token:

5 $$\begin{aligned} \psi _m = \left\{ \!\begin{array}{cl} \sigma _m > \sigma _n \forall (n \in \mathscr {M} \wedge n \ne m) &{}\rightarrow \psi _m + 1 \\ \text {otherwise} &{}\rightarrow \psi _m \end{array}\right. \end{aligned}$$

Further formalization is in Supplementary Information B and Fig. 2 illustrates the set of generative models.

To mirror the functional hierarchy of the visual processing stream, we parameterized the saliency maps ($\mathbb {P}_{b}$) within each model *m* with the hierarchical structure of the visual processing stream in humans. Early visual units encode low levels of saliency, such as edges, and contrast. Mid-level cortical centers encode proto-objects such as boxes, and cylinders, whereas later cortical centers encode object identifiers, motion, and faces. Thus, set $\mathscr {M}$ is a hierarchical set of models of the visual processing stream where lower-level models are presented in lower elements in the set (${m_i < m_{i+1}} \forall m \in \mathscr {M}$). Task-oriented saccade biases and inhibition of return are shared across all models in $\mathscr {M}$ as are thought to originate from higher cortical centers^24^.

### Validation and evaluation

To validate the generative model used for the analysis of the eye fixation data, we sought to understand its characteristics firstly in simulation, and then evaluate its performance on an external dataset where the internal cognitive state is known.

The simulation facilitates the probing of the generative models when both the bottom-up influences (i.e., the scene) and top-down influences (i.e., task-oriented saccade biases, and inhibition of return) are controlled for. This also facilitated the tuning of hyper-parameters $\beta$ and *T*.

The external dataset, one of visual search, facilitated the control of the scene, and hence bottom-up influences, through the explicit presentation of a stimulus whilst the eye patterns, and hence top-down influences, were left free. This facilitated the assessment of the generative models’ ability to recall ground truth cognitive internal states.

## Results

Following ethical and regulatory approvals, 42 patients were recruited culminating in 210 recordings; their characteristics are listed in Table 1. Recruited patients underwent a once-daily assessment of delirium through CAM-ICU^12^ with concurrent measurements of eye movements and the fixation on the scene, for 10 min daily until discharge from ICU. The data gathered was then used to infer the internal cognitive state of the patient through the generative model, dichotomized by the delirium status of the patient.

### Model with the highest reward reproduced the ground truth

To characterize the generative models, we first conducted a simulation exercise where we simultaneously created the scene and created the fixations and probed the general models for their robustness to noise and rapidity of change detection. This characterization also served to find the optimal value for hyper-parameter $\beta$. Supplementary Information C outlines the simulator and Supplementary Figures S4a and 4b illustrates the result of the simulation. A test of normality of the $\mathbb {P}_m$ demonstrated a highly non-normal (Kolmogorov–Smirnov test; $D=0.635$) cementing the requirement for sampling from the predictive distribution as per Eq. 3.

Two simulations were conducted, a constant simulation to identify model separation and a switch task for change detection. Model counts were significantly different from the simulated fixated environment and simulated, non-fixated, environment ($\chi ^2=190, p < 0.001, df=1$). There was no delay between the simulated fixation switch and the model’s response ($t(48)=4.8, p < 0.001$). This finding was consistent regardless of the fixation’s start position ($p = 0.98$) and irrespective of the location of the target ($p = 0.87$)

### Visual search task unveiled by the model with the highest reward

We next sought to demonstrate the generative models’ ability to generate plausible fixations under a fixed internal cognitive task. We used a pre-existing dataset from 26 participants undergoing a visual search task^27^.

The generative models were arranged hierarchically representing the functional connectivity of the processing stream of the participants with the addition of a center bias model due to the participants having fixed head positions. The model with the highest reward correctly identified the moment that the participant fixated on the visual search target ($\chi ^2=190, p < 0.001, df=4$). The model with the highest reward at time $t-1$ was also predictive of future fixation *f* and time *t* (ANOVA; $F=6.8, p < 0.001$).

**Table 2:** Results of the analysis of the generative models dichotomized by delirium status. Statistical significance testing using the $\chi ^{2}$ test between the tallied probabilities of delirium vs. non-delirium with the null hypothesis being of no difference demonstrates that there is a statistically significant difference across all functional visual processing modules between patients with and without delirium.

Generative Model	Delirium fixations	No delirium fixations	$\chi ^{2}$	df	*p* value
Early units	11,282	14,014	294	1	$< 2.2 \times 10^{-16}$
Mid units	11,282	14,011	295	1	$< 2.2 \times 10^{-16}$
Late units	13,729	16,441	244	1	$< 2.2 \times 10^{-16}$
Facial units	12,596	15,126	231	1	$< 2.2 \times 10^{-16}$

### Visual processing in acute delirium

Given our findings that the generative model set $\mathscr {M}$ is accurate in simulation, recalling visual attention behavior, and is predictive of future fixations, we analyzed the data gathered from patients in ICU undergoing task-free natural viewing.

Table 2 demonstrates the results of this analysis. We tallied the reward of each model which originates from a fixation originating from a recording that was labelled with the delirium status of the recording. The table demonstrates a statistically significant difference between the rewards of the models dichotomized by delirium status ($\chi ^2, p < 2.2 \times 10^{-16}$ for all models). This suggests that visual attention is markedly reduced between patients who have suffer from delirium compared to those who have not. Effect size is most striking for units in the early and middle of the hierarchy but is also present in late and facial units.

More specifically, the effect sizes are more substantial in the early and middle visual processing units. This finding suggests that the primary visual processing stages, which are responsible for the detection and basic interpretation of visual cues, are impaired in delirious patients. Additionally, similar disruptions are present in the middle units, which deal with the processing of proto-objects, indicating that the integration of visual information is also compromised.

Furthermore, the analysis also reveals differences in the late and facial recognition units. This indicates that not only are the basic and intermediate visual processing capabilities affected, but there is also a considerable impact on the higher-order visual processing abilities, which include the integration and interpretation of complex visual scenes and facial recognition.

## Discussion

Delirium is marked by acute disruptions in attention. Visual attention, a quantifiable and measurable subtype of attention, involves the selective processing of visual stimuli which we measured through eye-tracking using a custom eye-tracking apparatus that is suitable for use in critical care. This study focused on visual attention to understand the attentional impairments in delirium.

Altogether, our findings suggest a functional reduction in the working capacity of the ventral visual stream of patients suffering from delirium. This reduction is marked across the entire hierarchy of the visual stream including specialist areas such as the facial recognition unit.

Prior work on the understanding of the internal mechanisms highlighted that visual in-attention is a hallmark of acute delirium. Radiological investigations (CT, CT-PET, and MRI) have been heterogeneous but have broadly demonstrated reduced functional dysconnectivity between sensory processing and memory encoding units. These findings have been backed by EEG studies that suggest a reduction in high-order cortical activity in delirium; most importantly, the biggest discriminatory power for EEG seems to arise by the comparison of posterior and anterior electrodes. However, both radiological and electrophysiological techniques have failed at identifying the dysfunction of those areas and have to be inferred from the structure and counter function of studies of healthy individuals. Biochemical analysis of cerebrospinal fluid from patients suffering from delirium highlighted increased metabolic pressure suggesting restricted function at a neuronal level.

To understand the functional issues underlying visual attention in delirium, we reformulated the task of visual attention to fixation prediction and decomposed that task to account for bottom-up and top-down influences. This formulation led to a generative model that generates future fixation predictions that are conditional on past fixations and the scene. This facilitated the generation of multiple plausible fixations under different assumptions and the measurement of which generating process is most closely in line with viewing behavior. We initially validated the generative models in increasingly complex simulations to ascertain their properties. We then went further and validated them using a pre-existing dataset of visual search where we demonstrated accurate inference of the internal cognitive task to a highly statistically significant degree.

Lastly, and in answering our original question, we gathered data from 262 recordings from 42 patients in an ICU setting across two hospitals. In this experiment, we modeled visual attention using a similar computational hierarchy of the human visual processing cortex. We demonstrate that visual processing throughout the ventral visual stream has functionally reduced capacity compared to patients not suffering from delirium. The uniformity in the statistical significance across these different units of visual processing underscores the widespread nature of the impact that delirium has on the visual attention system. These results provide a quantitative basis for understanding the severity and extent of visual processing disruptions in delirious patients, restricted not only to the initial stages of visual attention but extending throughout the visual processing hierarchy.

This finding suggests that visual encoding and visual processing may play a role in the development of delirium, potentially preceding cognitive impairments. It provides valuable insights into the disease process and opens avenues for further research into the role of the ventral visual stream in acute delirium. Additionally, this finding has potential clinical implications, as interventions aimed at modifying the course of delirium can now consider the impact on visual processing, which can serve as a meaningful indicator of intervention effectiveness.

### Limitations and future work

While we have demonstrated the utility of our generative models in understanding the functional impairments in visual processing in delirium, there are several limitations to our study. Firstly, the generative models were informed by pre-existing data on the visual processing in humans, and thus the set of models ($\mathscr {M}$) was restricted to a set of 4 models. A larger set of models would represent a more granular inspection of the visual processing hierarchy. Secondly, the hyper-parameters for each model *m*, although informed by validation on an external dataset, could be markedly different in patients suffering from delirium and thus represent a form of possible distributional shift. Future work should focus on expanding the set of generative models to include more granular models of the visual processing hierarchy and to investigate the impact of distributional shifts on the performance of the generative models. Additionally, future work should focus on investigating the impact of different hyper-parameters on the performance of the generative models in patients suffering from delirium.

Also, while task-oriented saccade biases’ distributions have a myriad of shapes, we chose to model them as a minimum entropy distribution. Future work should focus on investigating the impact of different distributions on the performance of the generative models in patients suffering from delirium. This can be done by using eye-movement data from recordings of patients with delirium in this study. Lastly, a set of those saccade biases can be incorporated in the analysis in a combinatorial fashion but would require a more sophisticated model selection algorithm and a larger sample size. Lastly, we can also use a data-driven approach to jointly learn the structure and parameters of the simulated visual processing pipeline, which would make introspection even more impactful.

## Conclusion

The ventral visual processing stream of patients suffering from acute delirium is functionally restricted compared to patients who are not delirious. This implicates visual encoding as an upstream process prior to cognitive processing in the development of delirium. Further work is required to phenotype the deficits across sub-populations suffering from inflammatory, toxic, or hypoxic delirium.

### Supplementary Information

Supplementary Information.

## Data Availability

The code used to analyze the data is available upon request following appropriate regulatory and audit reviews. The patient data contains identifiable information and thus, unfortunately, cannot be shared due to ethical, regulatory, and protocol restrictions. Data and code for the simulation and the analysis of the visual search are freely available upon request by emailing a.al-hindawi@imperial.ac.uk.
