# Supplementary material for: A prospective multi-center study quantifying visual inattention in delirium using generative models of the visual processing stream
Source: Sci Rep. 2024 Jul 8;14:15698. doi: 10.1038/s41598-024-66368-4 (PMC11231180; doi:10.1038/s41598-024-66368-4)
Supplement: Supplementary file 1 — Supplementary Information. [file 41598_2024_66368_MOESM1_ESM.pdf]

A Patient Recruitment and Characteristics

The study was approved by the NHS HRA and REC (20/LO/0162) and registered on ClinicalTrials.gov (NCT04589169[15]). Eligibility Criteria were defined using the following criteria.

Inclusion Criteria

1. Age ≥ 18
2. Length of Stay ≥ 2 days
3. Estimated risk of delirium $\geq 20\%$, as predicted by E-PRE-DELIRIC

Exclusion Criteria

1. Lack of consent
2. Pre-existing Dementia
3. Visual Impairment, including dis-congruent eyes (convergent or divergent squint)
4. Inability for facial recognition to be carried out robustly
5. Inability to carry out CAM-ICU reliably

As delirium is a capacity-losing state, consent was sought from a next of kin in the first instance, or a nominated consultee in the second instance, should the patient be deemed lacking in capacity. Patients who regained capacity were re-approached for retrospective consent. Neither participants, their next of kin, or nominated consultees, receive monetary compensation in exchange for study enrollment.

Table 1 lists the patient characteristics recruited across the two sites. The two sites are balanced in important confounders relating to the severity of disease as measured by Acute Physiology and Chronic Health Evaluation II (APACHE-II) and risk of delirium.

Fig. A3 illustrates the flow of the patients in our study and how the data was analyzed using our generative models of the visual processing stream.

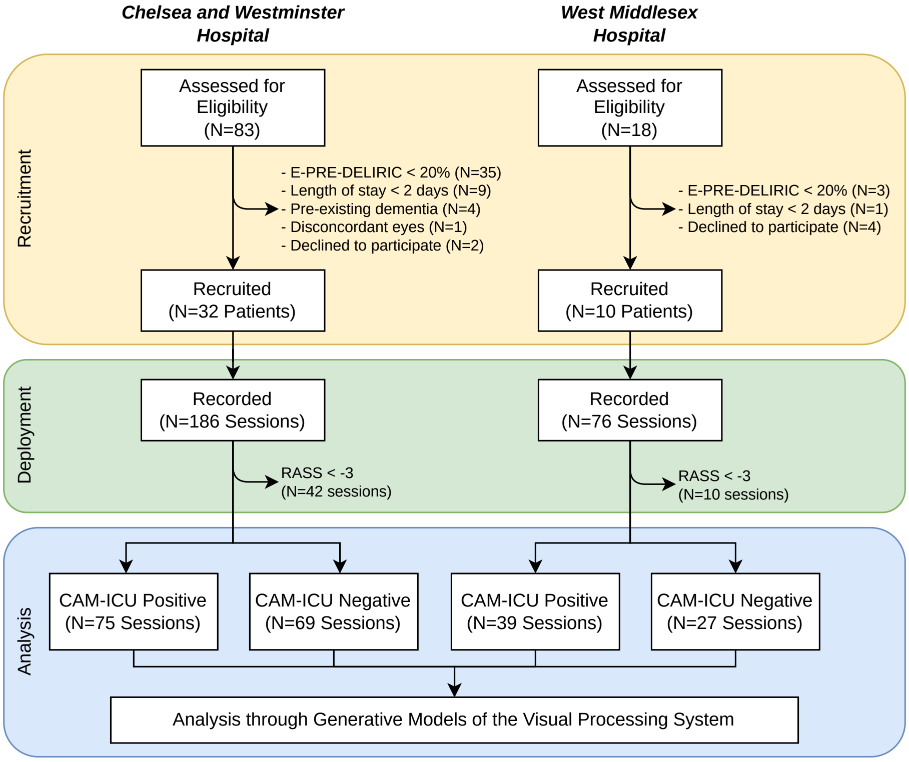

Figure A3. A diagram of the flow of patients and their recordings in the study and how the data was analyzed using our generative models of the visual processing stream.

B Generative Models

In this section, we formalize the architecture of a theory-of-mind approach using hierarchical models. Fig. 2 illustrates the architecture pictorially.

B.1 Overview

Multiple competing models are built each using a pipeline composed of *task-oriennted saccade biases*, *inhibition of return*, and *saliency* modules where the outputs are multiplied, element-wise.

The first module is a saliency model that, given a raw image of the scene from an ego-centric perspective, would output a probabilistic map of where fixations are likely to result given an intrinsic definition of saliency. Coupled with each saliency module is a simulation of eye gaze movement which is parameterized by the current fixation and consists of two sub-modules. The first sub-module, termed *saccade biases*, is a probability distribution that jointly encompasses the amplitude and direction of the next fixation given the location of the current fixation, which is often task-specific. The second module, *inhibition of return*, captures the statistical probability that the current fixation inhibits future fixations due to visual processing of the scene at that fixation. These two sub-modules constrain the saliency model into an area of the scene that the next fixation could plausibly reach given the ballistic nature of saccades. The result of each model module is thus a 2D map of possible fixations and their respective probabilities.

Given the list of fixations and their respective probabilities per model, a Monte-Carlo sampling module outputs the *correctness* of the model given the next gaze measurement from the participant fix_{t+1}. The *correctness* of the model is thus the average distance the simulation's predictions are from the next fixation point fix_{t+1}. However, due to the highly non-parametric distribution that results from the previous step, and of errors of measurement of the fixation point, simply outputting the density of the model under fixation fix_{t+1} would result in erroneous measurements to the central tendency of that non-parametric distribution. To alleviate this, the Euclidean distance between fixation fix_{t+1} and the sample from the set of simulated fixations is used instead of the Euclidean distance.

The result is the average distance for each model with the model that is most in line with fixation fix_{t+1} having the highest score. These scores are then converted to probabilities using a modified soft-max function.

B.2 Bottom-Up Influences

One of the inputs into the model is the scene image from an ego-centric perspective. This is used to calculate saliency, which is defined as a two-dimensional discrete probability mass function that places density in locations where fixations are expected. The mapping between the image and to probability mass function is heterogeneous in definition with multiple saliency models defining the mapping according to different metrics. For our proposed model, no restriction on the metrics is placed but a selection of saliency models should be coupled to potential tasks that the participants would be undertaking and highlight potential areas of interest in the scene.

B.3 Top-Down Influences

Saccade Biases A Task Oriented Saccade Bias (TOSB) is a joint distribution of the saccade's direction and amplitude and has been demonstrated to be task specific[26]. Rather than utilize pre-existing best-fit distributions that aim to cover the different tasks, we choose to not restrict the distribution of saccade biases and thus use the least informative distribution – a Gaussian distribution centered at the fixation with a $2°$ full-width-half-maximum. The input into this module is the saliency of this model and the output is a 2D probability map that constrains the saliency into a region where the eye is likely to saccade to next. Fig. 2 illustrates the distribution empirically chosen for this sub-module.

Inhibition of Return Once a participant's gaze has fixated on a scene location, they are not likely to look at that same location again shortly. This phenomenon has been termed inhibition of return and can be modeled by a decaying inverse Gaussian function over previous fixations[24].

B.4 Prediction Error

Given the map of fixations and their probabilities per model, a Monte-Carlo sampling module outputs the *correctness* of the model given the next gaze measurement from the participant fix_{t+1}. The *correctness* of the model encompasses the average distance the predictions are from the next fixation point fix_{t+1}. However, due to the errors of measurement of the fixation point, simply outputting the density of the model under fixation fix_{t+1} would result in erroneous measurements as an off-by-one pixel error would alter the density markedly. Secondly, the resulting distribution, given the pipeline of saliency $\rightarrow$ saccade bias $\rightarrow$ inhibition of return, is non-parametric (Kolmogorov-Smirnov test for all experiments below, $p < 0.05$) and thus an empiric distance metric is not defined. Thus, the Euclidean distance between fixation fix_{t+1} and a sampled fixation from the set of simulated fixations is used; the number of samples is empirically set to 10,000.

B.5 Competition

Given that each model is imbuing a simulation of an expected external observer, the model with the highest probability, and thus the lowest error is awarded in a Winner Takes All (WTA) strategy. This results in counts of each model which can then be compared across models.

C Simulation

To test whether the generative models' analysis correctly recalls ground truth data, a simulator was created. This was a scene composed of a 480× 270-pixel image of a red and blue circle aligned horizontally with a radius of 50px as illustrated in Fig. A4. Two models were created, one model that hypothesizes the viewer is looking at the red circle and one that hypothesizes that the viewer is looking at the blue circle.

Two experiments were undertaken, the first to ascertain if the generative models can reliably detect separation fixation origins and the other to see if they can detect a switch from one model to the next. To answer the first question, fixations were simulated to originate from the blue circle constantly while in the second experiment, the gaze was set to originate from the blue circle and then switch to the red circle. For each experiment, the fixation location is stochastically sampled from a Gaussian distribution centered on the circle with a standard deviation of half the radius. The generative models are then initialized with two saliency fields: one for the red circle, and one for the blue. Field of view is set to equal the field of view of a human; 210° horizontally and 150° vertically.

Saccade biases were modeled empirically as a Gaussian distribution with a full width at half maximum of 2°. Inhibition of return is modeled as per Eq. (2).

Fig. A5 demonstrates the results. The *constant* task in Fig. A5a is reliably and consistently classified with high probability. The second task, Fig. A5b, where the gaze would *switch* between two models is also detected with a sharp and immediate switch in probabilities. The *ripple* in probabilities is due to the added noise term to the gaze. This experiment also served as a method to grid-search a suitable value for β; a value of 50 was chosen for all subsequent experiments.

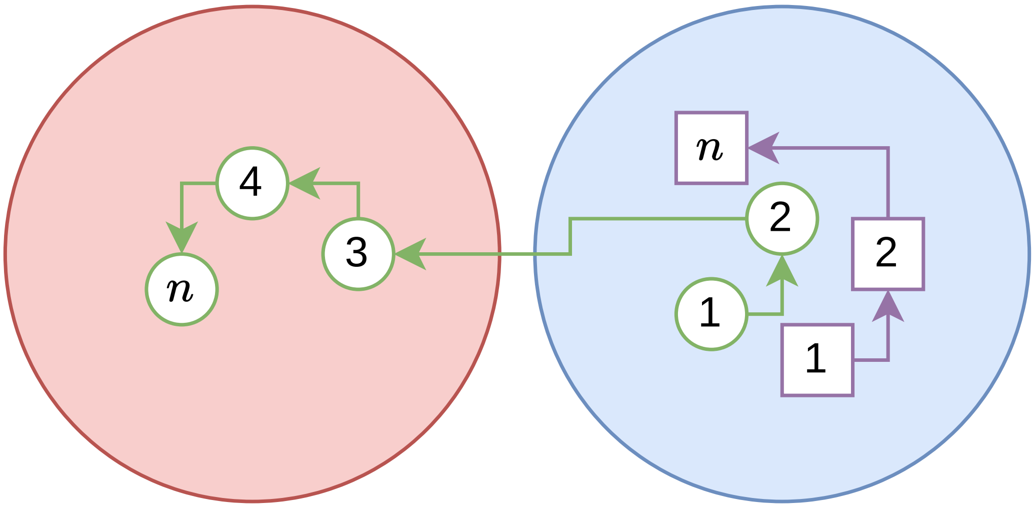

Figure A4. Illustration of one of the simulations results. The purple line demonstrates the first experiment where the fixation is set to originate from the blue circle. The green line demonstrates the second experiment where the fixation is set to originate from the blue circle and then switch to the red circle. The location of the fixations is randomised to originate from a Gaussian distribution centered on the circle with a standard deviation of half the radius. The location and size of the circles are also randomised.

a

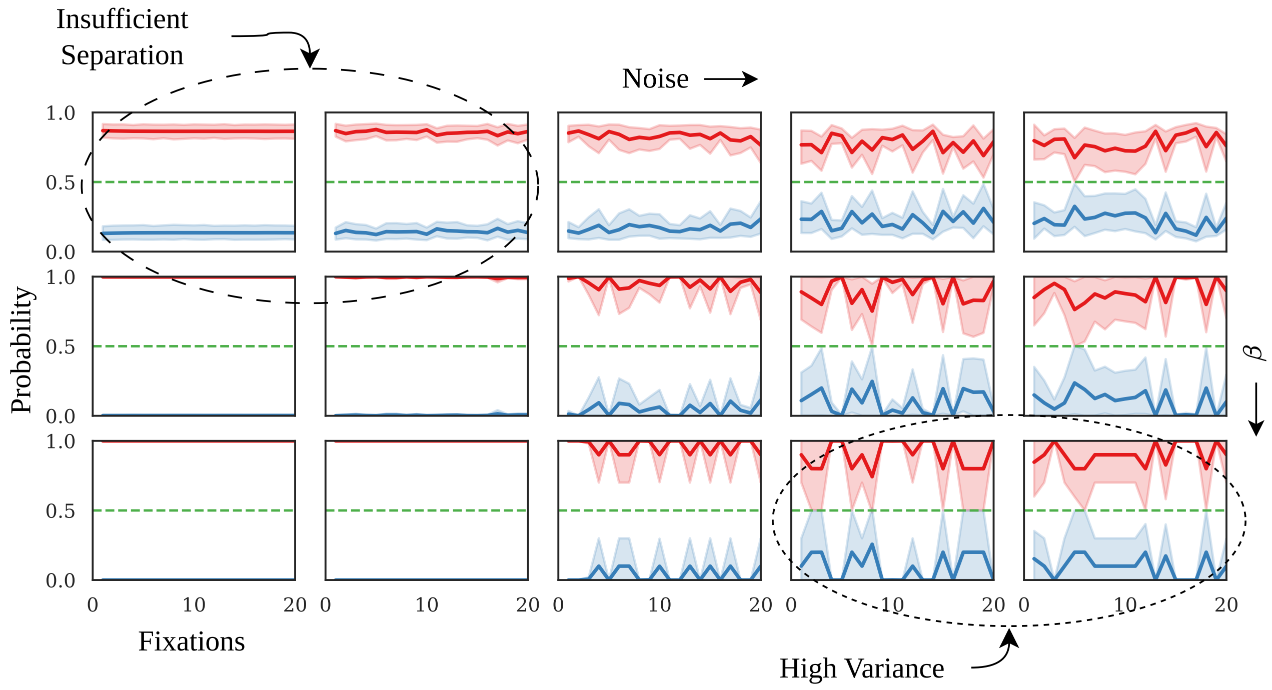

b

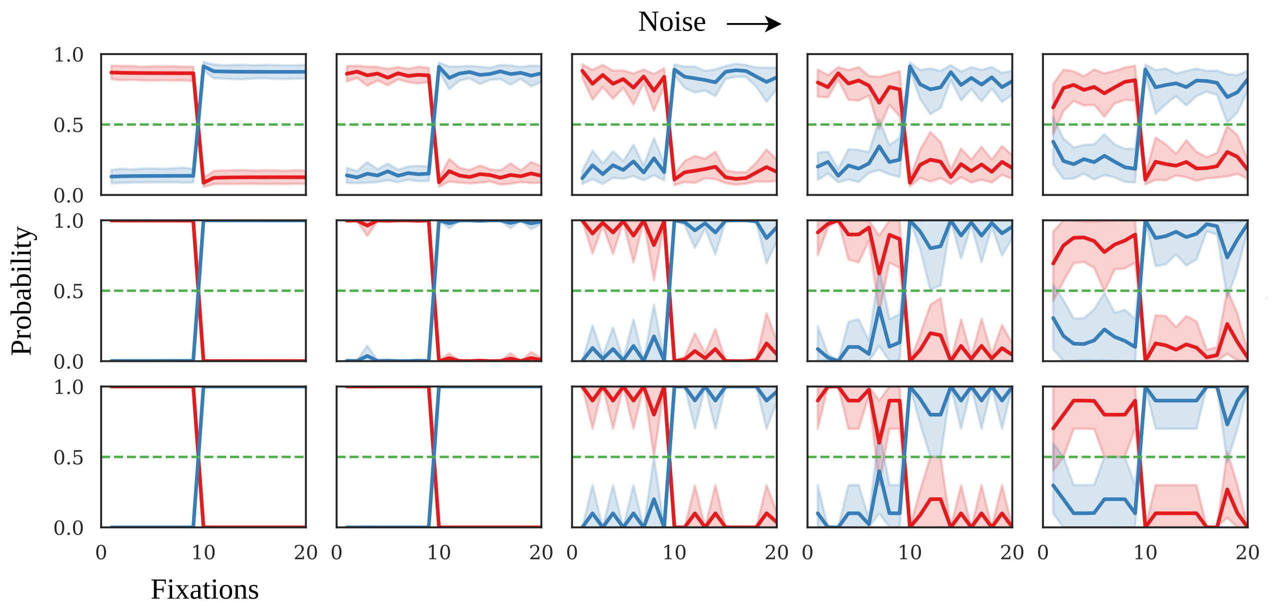

Figure A5. Simulation results for the proposed architecture that can probabilistically infer internal cognitive tasks. Two eye movement strategies are presented, a fixed strategy, and a switch strategy. Various β parameters are explored aiming to maximize the range of the softmax operator in Equation 4. Small values of β result in encroachment of the two simulations' probabilities while larger values maximize classification distance but lose sensitivity to small changes.

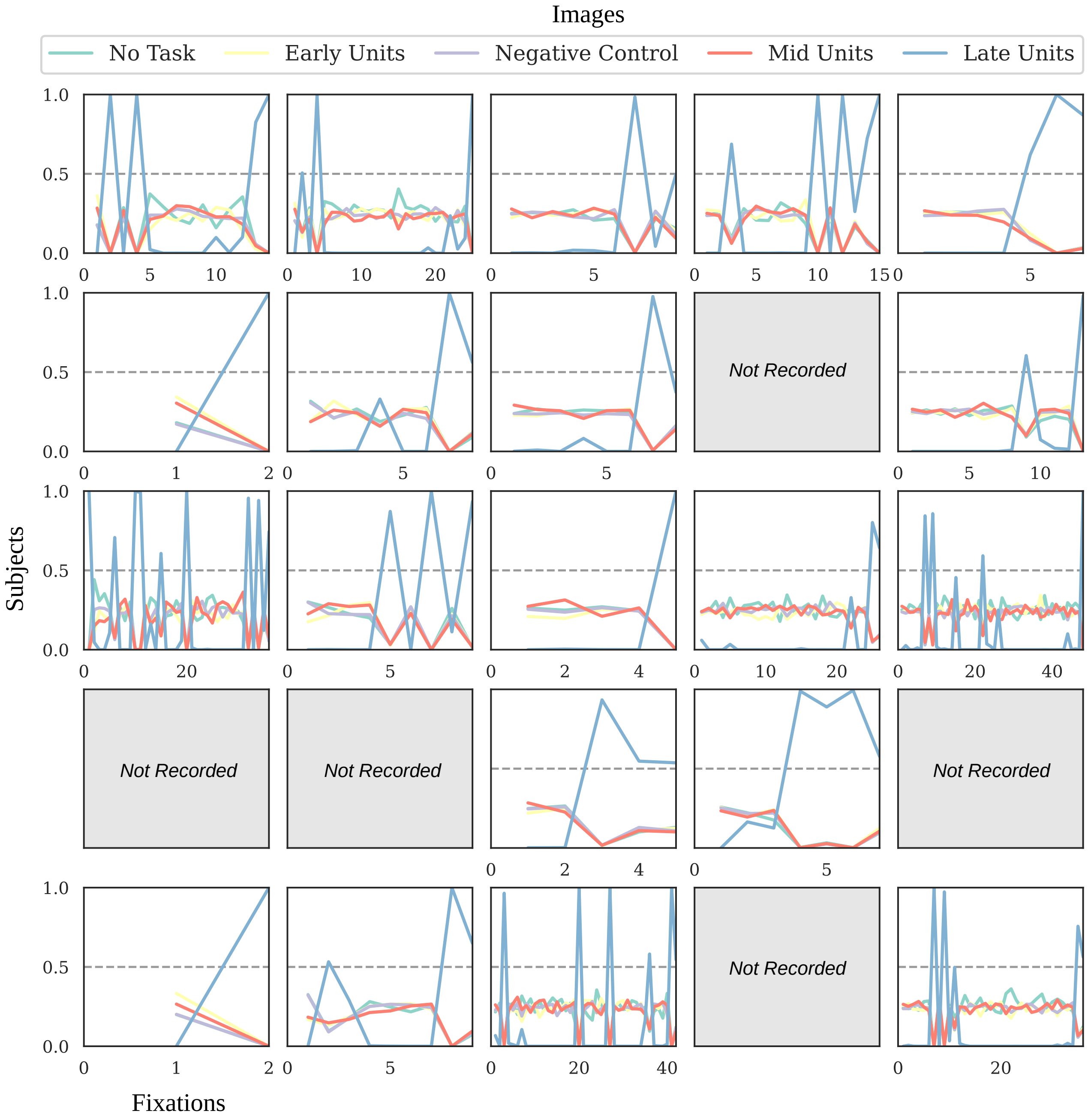

Figure A6. An exemplar set of 5 images with 5 subjects undergoing a visual search task. The blue line, which represents the model relating to later units, rises in competition to be the winner in all subjects when the object to be searched for is found; thus validating our architecture of estimating internal cognitive states using Eq. (1).

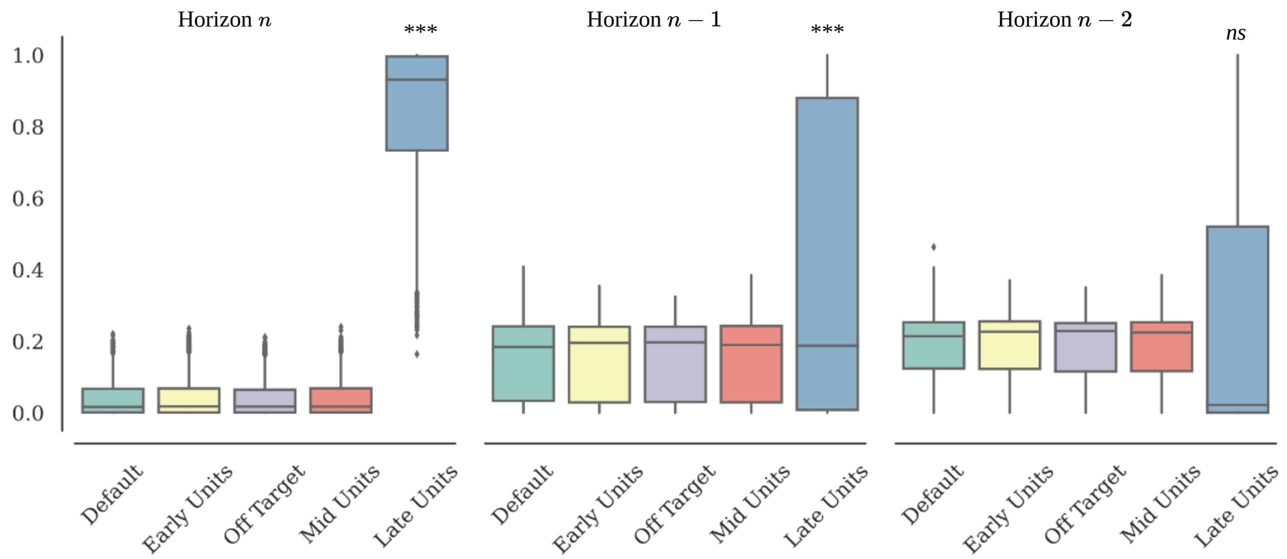

Figure A7. Prediction of the internal task at a given horizon where a horizon 0 is the current fixation, horizon $n-1$ is the previous fixation predicting the task undertaken by the current fixation. Statistical significance testing undertaken by ANOVA where $*** = p < 0.05$

D Visual Search Task

Zhang *et al*'s work on zero-shot invariant visual search produced a dataset of fixations from participants looking for Waldo - a cartoon character that is often hidden in a complex scene[27]. We use the *oracle* subset of this dataset which truncates the list of fixations across subjects such that the last fixation is the one closest to the location of Waldo. This serves as the ground truth - the last fixation's location is in sufficient proximity to the search target for the subjects to declare that they have located Waldo. The objective of this experiment is to ascertain the performance of the architecture in correctly classifying the fixations on Waldo during a visual search task where multiple switches between models could occur and the data originates from human participants in a controlled setting.

Five saliency models have been utilized that aim to explain the fixation strategies:

1. A object level 'late-unit' that places saliency over objects.

2. A synthetic *Off-Target* saliency that is the binary opposite of the ground truth supplied - this would represent *any* other search targets for all other objects

3. A saliency map representing early visual processing units that. This is operationalized by using Itti's saliency model aims to explain fixations in free-viewing in a biologically plausible manner[30]

4. A mid-unit 'proto-objects' saliency map that uses Spectral Residual Approach saliency model that uses aggregate image data from an information gain perspective to highlight proto-objects[29].

5. Lastly, a default 'center bias model' that places a two-dimensional Gaussian distribution on the center - this model is used to demonstrate control.

Similar to the simulation results in simulation, we model saccade biases as a Gaussian distribution with a full width at half maximum of 2° with a field of view as per the dataset[27]. Inhibition of return is modeled as per Eq. (2). Throughout the participant's search for Waldo, Fig. A6 illustrates that as the participant's fixation is closer to the target (Waldo), the model that predicts Waldo's probability increases. Due to the nature of the dataset, the last fixation is the one closest to the target and thus our proposed framework should robustly recreate this. Fig. A7 demonstrates that our proposed framework can reliably recreate the As the participant finds Waldo, the model with the Waldo prior then spikes and becomes the predominant winner.

Supplementary References

28. Ahmed Al-Hindawi and Marcela Vizcaychipi. Continuous Non-Invasive Eye Tracking for the Early Detection of Delirium on the Intensive Care Unit. Clinical Trial Registration NCT04589169, clinicaltrials.gov, December 2020.

29. Xiaodi Hou and Liqing Zhang. Saliency Detection: A Spectral Residual Approach. In *2007 IEEE Conference on Computer Vision and Pattern Recognition*, pages 1–8, June 2007.

30. Laurent Itti, Christof Koch, and Ernst Niebur. A model of saliency-based visual attention for rapid scene analysis. *IEEE Transactions on Pattern Analysis and Machine Intelligence*, 20(11):1254–1259, 1998.

31. Olivier Le Meur and Antoine Coutrot. Introducing context-dependent and spatially-variant viewing biases in saccadic models. *Vision Research*, 121:72–84, 2016.

32. Olivier Le Meur and Zhi Liu. Saccadic model of eye movements for free-viewing condition. *Vision Research*, 116:152–164, 2015.

33. Mengmi Zhang, Jiashi Feng, Keng Teck Ma, Joo Hwee Lim, Qi Zhao, and Gabriel Kreiman. Finding any Waldo with zero-shot invariant and efficient visual search. *Nature Communications*, 9(1):3730, September 2018.
